# Supplementary material for: Lipoxygenase Pathways in Diatoms: Occurrence and Correlation with Grazer Toxicity in Four Benthic Species
Source: Mar Drugs. 2020 Jan 19;18(1):66. doi: 10.3390/md18010066 (PMC7024367; doi:10.3390/md18010066)
Supplement: Supplementary file 1 [file marinedrugs-18-00066-s001.pdf]

Supporting Information

Lipoxygenase Pathways in Diatoms: Occurrence and Correlation with Grazer Toxicity in Four Benthic Species

Nadia Ruocco,[1,2] Genoveffa Nuzzo,[1] Giuliana d'Ippolito,[1] Emiliano Manzo,[1] Angela Sardo,[1] Adrianna Ianora,[2] Giovanna Romano,[2] Antonella Iuliano,[3] Valerio Zupo[2], Maria Costantini,[2] Angelo Fontana[1*]

[1] Consiglio Nazionale delle Ricerche, Istituto di Chimica Biomolecolare, Via Campi Flegrei 34, 80078, Pozzuoli, Napoli, Italy.

[2] Department of Marine Biotechnology, Stazione Zoologica Anton Dohrn, Villa Comunale, 80121, Napoli, Italy.

[3] Consiglio Nazionale delle Ricerche, Istituto per le Applicazioni del Calcolo "Mauro Picone", Via Pietro Castellino 111, 80131 - Napoli.

Fig. S1 GC-MS profile of *N. shiloi* extract with indication of PUAs.

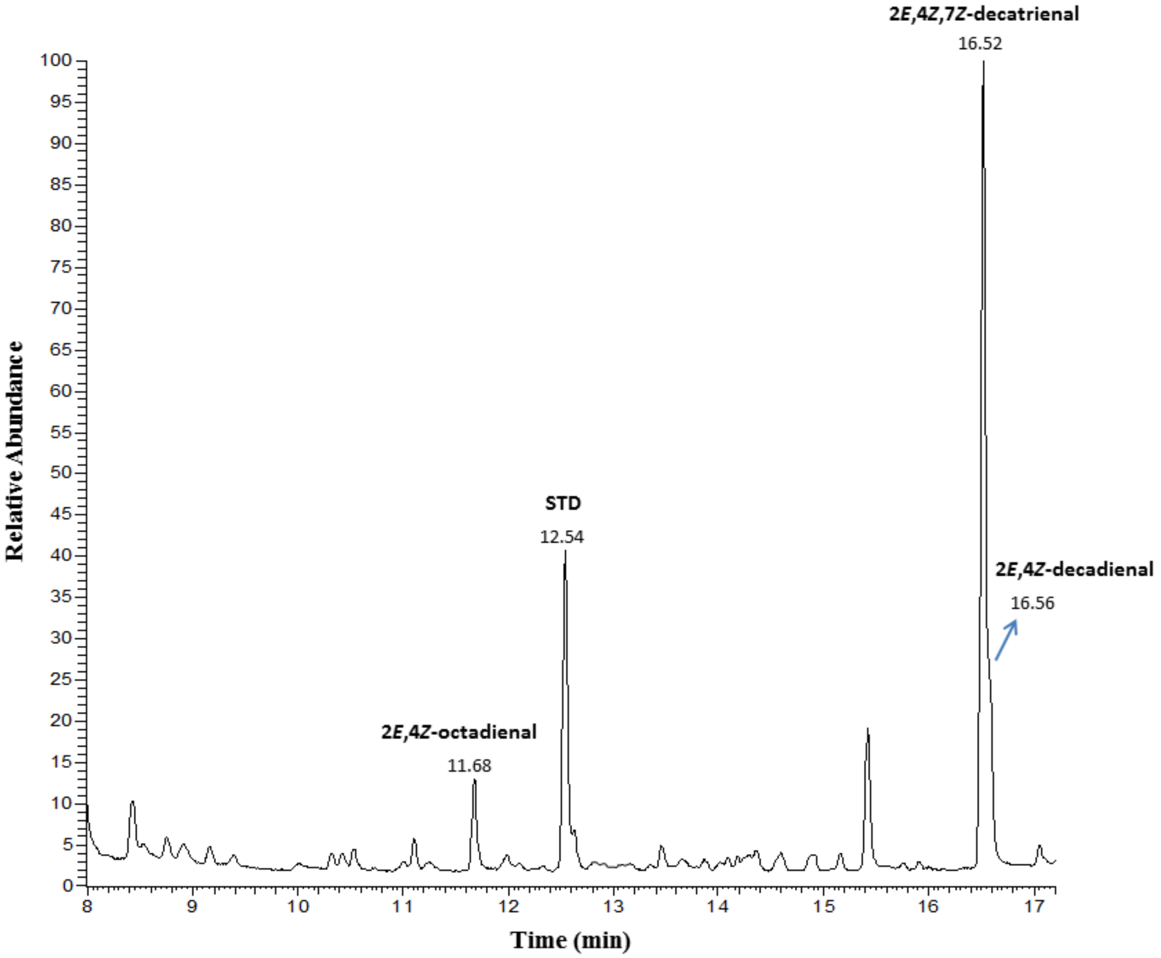

Fig. S2 Bioassays reported in Ruocco *et al.*, 2018, 2019. (**a**) Percentage of abnormal plutei spawned from sea urchins fed with *U. rigida* (CTRL) and the four benthic diatoms (TREATED); (**b**) Examples of normal and malformed plutei observed in CTRL and TREATED conditions, respectively.

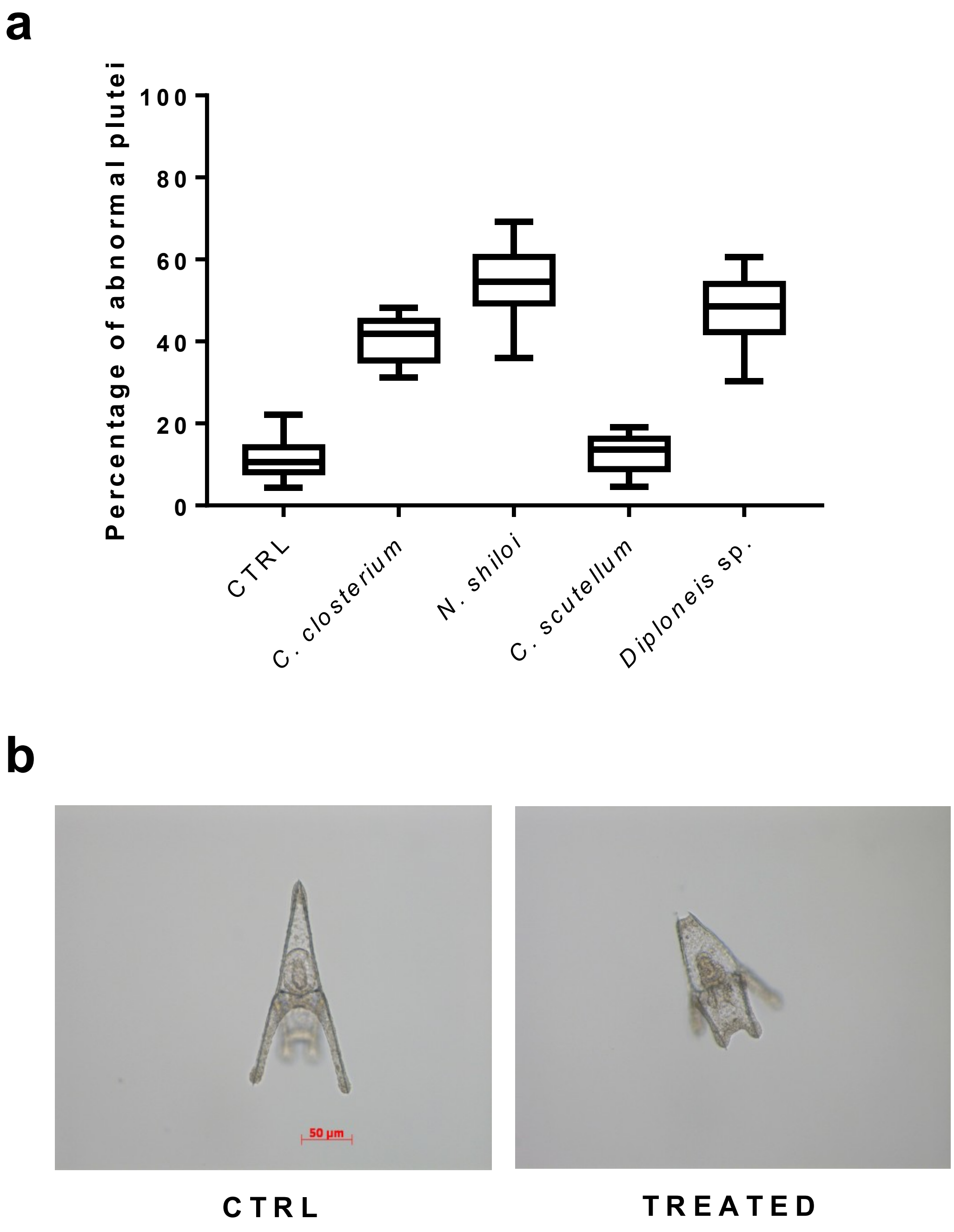

Fig. S3 Effects on embryos of sea urchin *P. lividus* by feeding experiments with the four benthic diatoms. (**a**) Data of plutei survival (n =17) as reported in Ruocco *et al.*, 2018, 2019; (**b**) Best three fits of plutei survival for the 17 experiments.

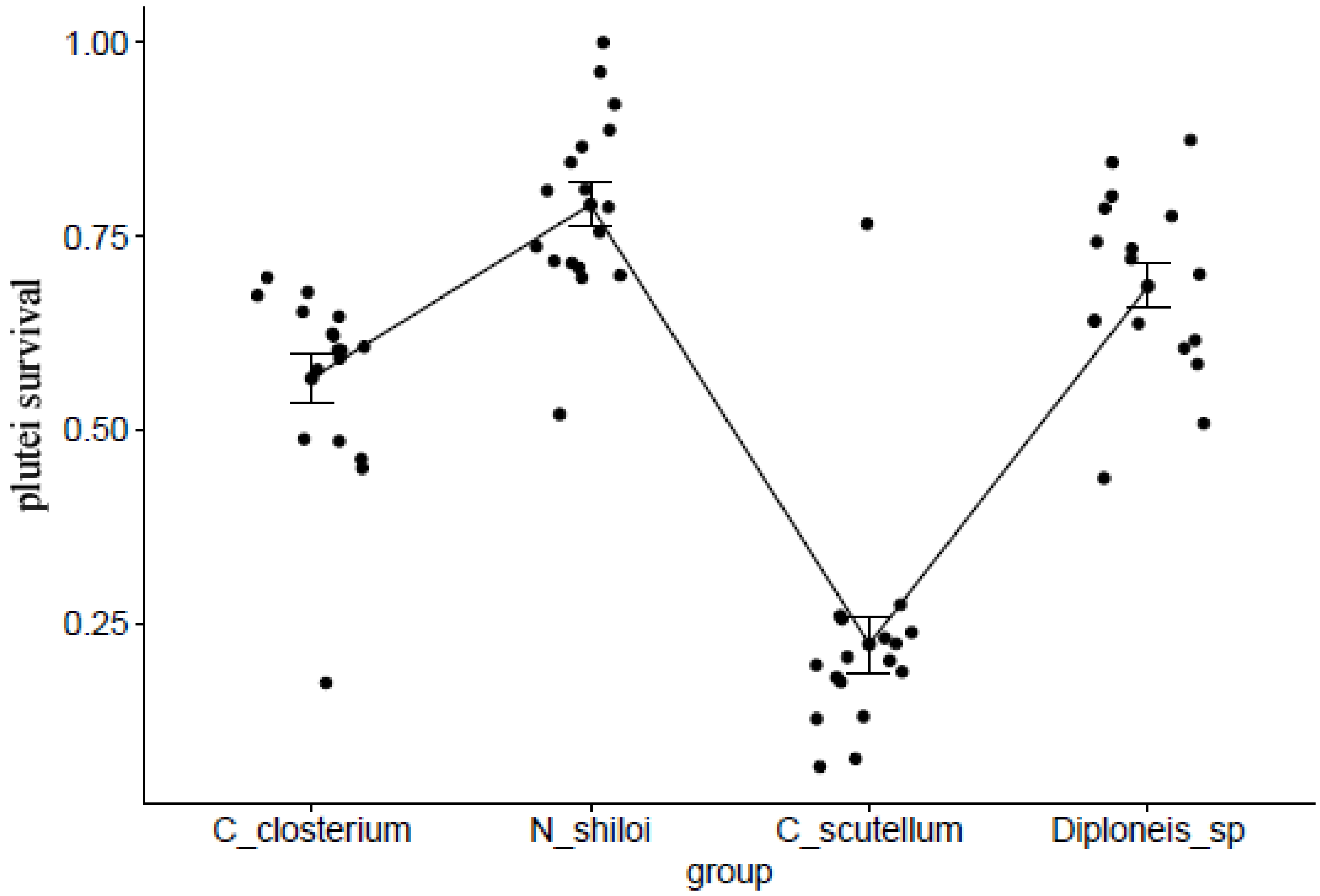

group	n	mean	sd	min	Q1	median	Q3	max	sem
C_closterium	17	0.567	0.127	0.173	0.488	0.603	0.646	0.697	0.0308
C_scutellum	17	0.223	0.152	0.065	0.175	0.202	0.238	0.766	0.0369
Diploneis_sp	17	0.686	0.117	0.438	0.616	0.701	0.776	0.874	0.0284
N_shiloi	17	0.791	0.117	0.520	0.715	0.788	0.866	1.000	0.0284

Table S1 Post-hoc analysis by Multiple Comparisons of Means (Tukey Contrasts) for distribution of oxylipins in the four diatom species.

group	n	mean	sd	min	Q1	median	Q3	max	sem
C_closterium	3	0.321	0.072	0.267	0.280	0.293	0.347	0.402	0.041600
C_scutellum	3	0.100	0.009	0.090	0.097	0.104	0.105	0.106	0.005200
Diploneis_sp	3	0.003	0.001	0.002	0.002	0.002	0.003	0.004	0.000577
N_shiloi	3	0.907	0.083	0.841	0.861	0.881	0.940	1.000	0.047900

Table S2 Post-hoc analysis by Multiple Comparisons of Means (Tukey Contrasts) for distribution of FAHs in the four diatom species.

group	n	mean	sd	min	Q1	median	Q3	max	sem
C_closterium	3	0.284	0.042	0.237	0.267	0.297	0.307	0.317	0.024200
C_scutellum	3	0.199	0.008	0.190	0.195	0.200	0.203	0.206	0.004620
Diploneis_sp	3	0.011	0.001	0.010	0.010	0.011	0.011	0.012	0.000577
N_shiloi	3	0.955	0.040	0.921	0.933	0.945	0.972	1.000	0.023100

Table S3 Data used for building up of the ternary diagram.

Species	rep	Oxylipins	FAHs	BioActivity
C_closterium	rep1	0.2929803	0.3168820	0.6026012
C_closterium	rep2	0.4019410	0.2372503	0.5780347
C_closterium	rep3	0.2668113	0.2974966	0.6213873
N_shiloi	rep1	0.8805875	0.9999997	0.8092486
N_shiloi	rep2	0.9999957	0.9214978	0.8453757
N_shiloi	rep3	0.8405334	0.9445906	0.8656069
C_scutellum	rep1	0.0902770	0.1904850	0.2239884
C_scutellum	rep2	0.1039217	0.2055774	0.1878613
C_scutellum	rep3	0.1064433	0.2004594	0.2312139
Diploneis_sp	rep1	0.0020236	0.0104364	0.5852601
Diploneis_sp	rep2	0.0039663	0.0122832	0.6156069
Diploneis_sp	rep3	0.0022260	0.0105300	0.6401734

Table S4 Oxylipins (LOFAs and PUAs) found in the four diatoms under analysis with indication of the specific enzymatic activity and chemical structures.

SPECIES	ENZYME	CHEMICAL STRUCTURE
C. closterium		
**14,13-EHETE**	EPA:15-LOX	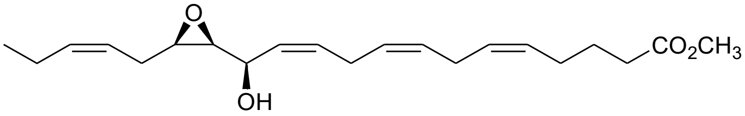
**15-HEPE**	EPA:15-LOX	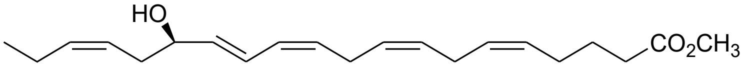
**16,15-EHDPE**	DHA:17-LOX	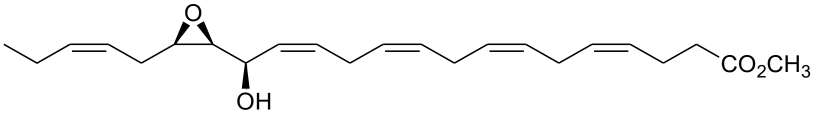
N. shiloi		
**9,11-EHHDE**	HTE:9-LOX	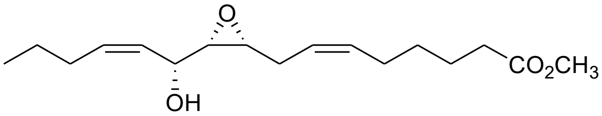
**11,10-EHETE**	EPA:11-LOX	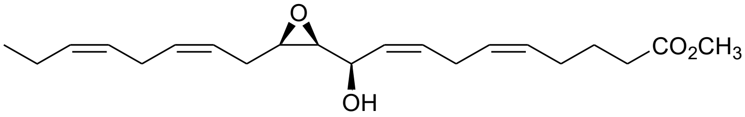
**11-HEPE**	EPA:11-LOX	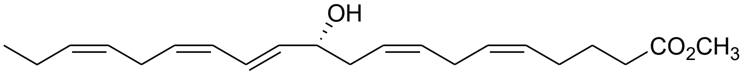
**9-KHME**	HDE:oxygenase	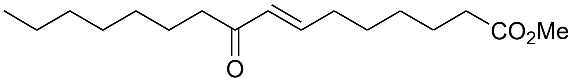
**9-HHME**	HDE:oxygenase	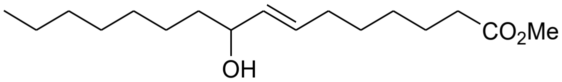
**2*E*,4*Z*-octadienal**	HTE:9-LOX	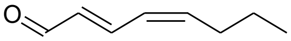
**2*E*,4*Z*,7*Z*-decatrienal**	EPA:11-LOX	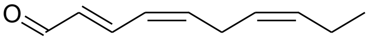
**2*E*,4*Z*-decadienal**	AA:11-LOX	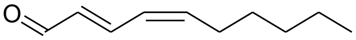
C. scutellum		
**14,13-EHETE**	EPA:15-LOX	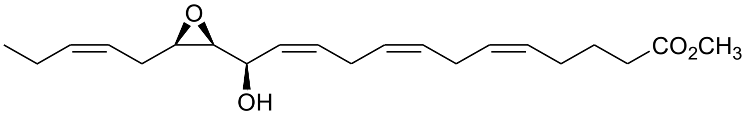
**15-HEPE**	EPA:15-LOX	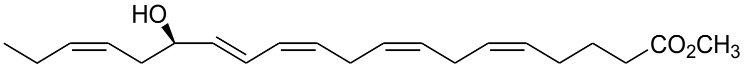
